# HCV-Induced Oxidative Stress: Battlefield-Winning Strategy

**DOI:** 10.1155/2016/7425628

**Published:** 2016-05-12

**Authors:** Khadija Rebbani, Kyoko Tsukiyama-Kohara

**Affiliations:** Department of Animal Hygiene, Joint Faculty of Veterinary Medicine, Kagoshima University, Kagoshima 890-0065, Japan

## Abstract

About 150 million people worldwide are chronically infected with hepatitis C virus (HCV). The persistence of the infection is controlled by several mechanisms including the induction of oxidative stress. HCV relies on this strategy to redirect lipid metabolism machinery and escape immune response. The 3*β*-hydroxysterol Δ24-reductase (DHCR24) is one of the newly discovered host markers of oxidative stress. This protein, as HCV-induced oxidative stress responsive protein, may play a critical role in the pathogenesis of HCV chronic infection and associated liver diseases, when aberrantly expressed. The sustained expression of DHCR24 in response to HCV-induced oxidative stress results in suppression of nuclear p53 activity by blocking its acetylation and increasing its interaction with MDM2 in the cytoplasm leading to its degradation, which may induce hepatocarcinogenesis.

## 1. Introduction

Hepatitis C chronic infection is a major public health issue and continues to make annually around 500.000 deaths due to hepatitis C-related liver diseases [1]. Hepatitis C virus (HCV) is a positive single stranded RNA hepacivirus (family Flavivirus, genus* Hepacivirus*); the genome size is 9.6 kb, flanked by highly conserved untranslated regions (UTRs) at 5′ and 3′ ends and encoding a large polyprotein of 3010 amino acids, that will be co- and posttranslationally processed into 3 structural (core and envelope proteins E1 and E2) and 7 nonstructural (P7, NS2, NS3, NS4A, NS4B, NS5A, and NS5B) mature proteins. The translation of this polyprotein is initiated by an internal ribosomal entry site (IRES) harbored in the 5′-UTR [2]. HCV has a hepatic tropism and a cytoplasmic life cycle; however, it was established that HCV is able to initiate an abortive cycle in dendritic cells (DCs) and B lymphocytes [3, 4]. E1 and E2 envelope proteins play an important role in HCV-hepatocyte attachment and entry and are involved in direct cell surface interactions with cellular receptors. Several cell receptors were identified as being potential HCV cell entry to the cell, mainly transmembrane lectins as dendritic cell-specific intercellular adhesion molecule-3-grabbing nonintegrin (DC-SIGN) and its liver and lymphatic endothelium homologue (L-SIGN), scavenger member 1 receptor-class B (SR-BI), tight junction proteins such as claudin-1 (CLDN-1) and occludin (OCLN), cluster of differentiation 81 (CD81) protein, and, recently, the very-low-density lipoprotein receptor (VLDLR) [5, 6]. The entry into the host cell is followed by uncoating and release of the viral genome, translation of viral proteins, replication of the viral genome, and assembly and secretion of virions. All these events take place in the cytoplasm of the host cell. Uncoating allows exposure of the viral genome to cellular mechanisms. The viral RNA serves first as messenger RNA (mRNA) for viral protein translation. This step occurs in the endoplasmic reticulum (ER). After translation of proteins required for viral replication, the viral RNA serves as a template for synthesis of positive single stranded RNA progeny, in association with intracellular membranes. Although data on the late stages of viral replication is limited, it is currently recognized that the assembly and maturation of viral particles occur in the endoplasmic reticulum and in the Golgi apparatus, to be excreted afterward in the extracellular medium and the bloodstream [7, 8]. This scheme lines the direct involvement of HCV proteins in oxidative stress induction. The exact mechanisms triggering the establishment of chronic infection remain little known, although several mechanisms were propounded, mainly the disruption of interferon (IFN) response, inhibition of DCs and natural killers (NK) cell functions, induction of autophagy, and promoting chronically infected cells throughout modulation of complement system [5, 7, 9]. Several evidences suggest a strong association between HCV chronic infection and metabolic disorders such as steatosis, insulin resistance, and iron load disregulations causing “a specific HCV-associated dysmetabolic syndrome (HCADS)” as introduced by Lonardo in a correspondence to the Editor of Hepatology [10–12], and all these pathologies have been related, in one way or another, to the oxidative stress. Indeed, a complex interconnection between HCV, oxidative stress, insulin resistance, and steatosis exists: HCV-induced oxidative stress affects the insulin signaling in hepatocytes after the dephosphorylation of AMP-activated kinase via activation of protein phosphatase 2A (PP2A), an inhibitor of Akt protein. Insulin resistance in turn may contribute to steatosis by inducing sterol regulatory element binding transcription factor 1 (SREBF1) that will lead to an increase in fatty acid biosynthesis. Likewise, steatosis might exacerbate both insulin resistance and oxidative stress and accelerates the progression of fibrosis [13–16].

Redox signaling is primordial for the proper functioning of the cell, and the generation of reactive oxygen species (ROS) or reactive nitrogen species (RNS) could be a normal process in the life cycle. Nonetheless, the transiently or chronically enhanced production of ROS may disturb the cellular metabolism and its regulation and damage cellular constituents leading to a situation of oxidative stress, defined simply as an imbalance in the ratio of oxidant/antioxidant particles [17]. This imbalance is often due to an increase in oxidant particles production (ROS/RNS) and a deficiency in antioxidant defense, via either deregulation of enzymatic systems (superoxide dismutase (SOD), glutathione peroxidase/reductase (GPX1/GSR), glutathione transferase (GST), thioredoxin reductase (TXNR), catalase (CAT), heme oxygenase (HMOX), peroxiredoxin (PRDX), and paraoxonase 1 (PON1)) or decrease in antioxidant (vitamin C/E, glutathione, carotenoids, flavonoids, transferrin, albumin, bilirubin, and uric acid) [18, 19]. In the liver, ROS/RNS species including superoxide anions (O_2_^•−^), hydrogen peroxide (H_2_O_2_), hydroxyl radicals (HO^•^), nitric oxide (NO), nitrogen dioxide (NO_2_), and nitrate (NO_3_) are endogenously produced mainly by mitochondria, though other sources could be strongly involved in oxidative stress induction, particularly endoplasmic reticulum, via cytochrome P450 metabolism [19–22]. They could directly interact with biological molecules including proteins, lipids, and DNA or induce hepatocytes and other liver cells damage via fibrosis, apoptosis, or cell necrosis. Indeed, the activation of stress-induced signaling pathways, mainly mitogen-activated protein kinases (MAPK) and nuclear factor kappa B (NF-*κ*B) pathways, modulates protein expression and exposes hepatocytes to further oxidative stress [18, 23, 24].

## 2. Oxidative Stress and Hepatitis C Virus

Based on animal models and culture systems studies, it was shown that HCV expressed proteins seem to impair directly the mitochondrial respiratory chain through an overproduction of ROS, which alter both mitochondria's structure and function of infected hepatocytes [20, 24, 25]. Almost all HCV proteins were involved in the mitochondrial oxidative stress, with a focus on core and NS5A proteins as the main oxidative stress inducers, in opposition to the idea stating that a viral proteins accumulation is needed to induce oxidative stress [19].

Recently, a review from Garofalo et al. discussed the interaction between mitochondrial raft-like microdomains and disialoganglioside GD3 in the regulation of cell apoptosis. GD3 seems to enhance ROS overproduction, leading to an activation of cytochrome c- (Cyt c-) dependent caspase 3 (CASP3), that could be enhanced by the depletion of glutathione [26].

Likewise, it has been admitted that oxidative stress arises from the dysregulation of calcium signaling in the ER/mitochondria junctions [27]. The evident colocalization of core protein with the mitochondria-associated ER membranes (MAMs) might explain the eventual interaction between HCV core protein with subcellular organelles (mitochondria, ER, and MAMs structures) through modification of calcium redistribution. Indeed, it has been shown that the Ca^2+^ influx regulates the mitochondrial metabolism and MAMs structures ensure the transition of Ca^2+^ ions stored in the ER to the mitochondria, which leads to a reprise of homeostasis or a launching of apoptosis process via Fas ligand pathway. MAMs structures have been recently reported to play an essential role in cell response to stress and controlling HCV replication and persistence. The functional depletion of MAMs associated proteins such as sigma-1 receptor (S1R) could modulate both the viral cell cycle and the host response to stress [19, 24, 26, 28]. However, less is known about the role of these structures in the maturation and infectivity of HCV particles.

At another level, the growing fact that mitochondrial dynamics and mitophagy may be affected directly by HCV sheds the light on a new track of HCV immune escape and persistence strategies [29]. In normal cells, the mitochondrial fission, fusion, and mitophagy are tightly monitored in a control quality loop. However, as previously described, HCV promotes Drp1 phosphorylation and its mitochondrial translocation, which leads to mitochondrial fission promotion and the induction of Parkin-dependent mitophagy. These events attenuate apoptosis of HCV-infected cells and support virus secretion, and immune escape and persistence [30, 31].

## 3. HCV-Induced Oxidative Stress and Liver Diseases

During acute infection, the incubation phase during the first two months of infection is characterized by high rates of HCV viral load in the serum. The increase in enzyme levels transaminases declares the beginning of an acute phase of infection that lasts a few months before clearance of the virus. In 70–75% of cases, immune response fails to eliminate the virus during the acute phase, and the infection persists. The chronic phase is generally characterized by a stable viral load, relatively normal transaminase levels, and an inflammatory microenvironment [7, 8].

Currently, the growing evidence that HCV-induced oxidative stress significantly contributes to hepatic disease has been supported by several studies on the correlation between markers of oxidative stress and those of liver injury, in particular, alanine aminotransferase (ALT) and aspartate aminotransferase (AST) which are the main seromarkers of liver disease [32, 33]. The vicious circle controlled by HCV and redox-oriented is commonly activated in major forms of chronic liver disease and plays a critical role in hepatic fibrogenesis and carcinogenesis. During the liver disease course, Kupffer cells trigger inflammation process, and it has been shown that HCV modulates inflammatory responses by inducing interleukin (IL)-1*β* overproduction and secretion by Kupffer cells and that oxidative stress markers have been shown to correlate with severity of inflammation. Besides, it was supported that the induction of immune response by HCV led to the activation of Kupffer cells, which in its turn contribute to the profibrotic cytokines and ROS release, though less is known about the exact mechanisms triggering Kupffer cells-induced oxidative stress and liver damage [24, 34].

Damaged hepatocytes release ROS in the extracellular environment, leading to the activation of hepatic stellate cells (HSCs). In addition to their role in collagen synthesis, these cells were reported as a supplementary source of ROS during fibrosis process. A series of studies have demonstrated that the activation of nicotinamide adenine dinucleotide phosphate (NADPH) oxidase in Kupffer cells and HSCs drives the production of ROS, leading to hepatocytes sensitization to fibrogenic and protumorigenic states [18, 35–37].

Unlike hepatitis B virus (HBV), HCV is not considered as a direct oncovirus, does not possess canonical oncogenes, and is unable to integrate into the host genome. However, the chronic inflammatory episode is the main path leading to HCC development [35–40]. Cytokines and ROS released by either nonparenchymal liver cells (Kupffer cells and HSCs) or immune effector cells (macrophages, mast cells, DCs, and NK cells) recruited during inflammatory response are major mediators of protumorigenic state of hepatocytes. Activated Kupffer cells produce tumor necrosis factor-*α* (TNF-*α*) which plays a central role in mediation of proinflammatory immune response and recruitment of blood cells to the site of liver injury. Although a heap of data was published on the role of TNF-*α* in liver disease, when and how this mediator induces intracellular apoptotic or antiapoptotic pathways are still nonanswered questions. The establishment of the chronology in the loop involving ROS release, oxidative stress occurrence, suppression of JAK/STAT pathway, TNF-*α* production, and induction of NF-*κ*B pathway might enlighten many dark spots on the oxidative stress-induced HCC mechanism [18, 19, 41, 42].

Although the link between oxidative stress and TNF-*α* production is well established in alcoholic liver disease, it is still not clearly determined in case of HCV-associated liver disease [42].

## 4. DHCR24 in Response to Oxidative Stress: An Ally or a Belligerent?

The 3*β*-hydroxysterol Δ24-reductase (DHCR24) is one of the newly discovered host markers of oxidative stress. The gene coding DHCR24 is located on chromosome 1 (1p33-1p31.1), spans 46.4 kb, and comprises 9 exons and eight introns [43]. Rare mutations (E191K, N294T, K306N, and Y471S) in the DHCR24 gene result in an autosomal recessive disease called desmosterolosis that is characterized by elevated levels of desmosterol in the plasma, liver, and kidneys, developmental malformations, and neuropsychological alterations. It is acknowledged that DHCR24 plays a crucial role in maintaining cellular physiology via the regulation of cholesterol synthesis [44, 45] by catalyzing the conversion of desmosterol to cholesterol in the post-squalene cholesterol biosynthesis pathway. Description of its function as a flavin adenine dinucleotide- (FAD-) dependent oxidoreductase was behind the discovery of its pivotal role in stress response. Furthermore, the ER localization of DHCR24 corroborates the assumption of its direct involvement in oxidative stress response, and this localization changes under oxidative and oncogenic conditions [44]. Throughout its multifunctional backbone, DHCR24 exerts a modulating function in the prevention of stress-induced apoptosis when it is reexpressed at high levels and may exert an antioxidant role via scavenging of ROS [37, 44, 46].

**Figure 1:**
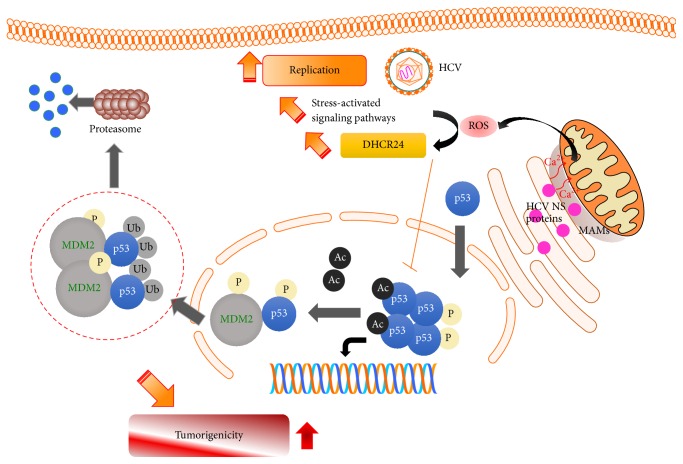
HCV-induced oxidative stress and HCC inception. HCV nonstructural (NS) proteins induced oxidative stress throughout disturbance of mitochondrial metabolism. The generation of ROS activates stress-induced signaling pathways that enhance the overexpression of DHCR24, a pivotal protein for HCV replication and HCC induction. HCV: hepatitis C virus, HCC: hepatocellular carcinoma, ROS: reactive oxygen species, DHCR24: 3*β*-hydroxysterol Δ24-reductase.

Several studies by our group showed that HCV possess a responsive region in the promoter region of DHCR24, in addition to being the binding site of the transcription factor Sp1 recruited in response to oxidative stress (Figure 1). Further studies showed that this specific region is highly controlled by epigenetic mechanisms since it locates on a CpG island on the promoter region of DHCR24 gene [47–49].

HCV-induced oxidative stress engenders an aberrant cholesterol trafficking and lipid metabolism dysregulation, leading to hepatic fibrosis and progression toward end-stage liver disease, ultimately hepatocellular carcinoma (HCC) which is the most frequent primary liver tumor [50]. The molecular epidemiology of HCC has been characterized by a singular variability between geographic regions depending on several factors, especially risk factors prevailing in each region. In the far eastern region, the region with the highest prevalence of HCC, the prevailing risk factors are HBV/HCV chronic infections [51]. By determining the region-specific molecular context of HCC, many questions could be resolved and many advances in the therapeutic approaches could be achieved. Though remarkable progress has been achieved in cancer molecular studies, mechanisms triggering hepatocarcinogenesis remain poorly known. Initiation of tumoral process needs, typically, a promoting agent which could be viral proteins themselves or viral-induced alterations that could lead to the liver tumorigenesis. Altered hepatocytes with the highest proliferative potential are the origin of the malignancy [52]. Several pathways were propounded to be altered in liver carcinogenesis. However, since 1989, the most studied gene in the class of tumor suppressor genes has been TP53, the gene encoding the phosphoprotein p53 triggering cell cycle checkpoints, apoptosis, senescence, and DNA repair, by regulating expression of several target genes [53]. P53 pathway consists of a network of proteins that are induced in response to a signal of intrinsic or extrinsic stress. The second most studied mechanism is related to the regulation of P53 by E3 ubiquitin protein ligase (MDM2) protein. This oncoprotein targets, with high specificity, the P53 protein by binding to its N-terminal transactivator domain and induces its proteasomal degradation. MDM2 gene expression is induced by the wild form of the protein P53 itself through its binding to the first intronic region of MDM2 gene: the P53/MDM2 interaction is a feedback loop regulating the concentration of active p53 [54, 55]. Our data showed that DHCR24 is specifically expressed on the surface of HCV-HCC cell lines and that high levels of anti-DHCR24 antibodies were detected in the sera of patients with HCV-related HCC. These data demonstrate that overexpression of DHCR24 in HCC is specifically induced by HCV. Furthermore, overexpression of DHCR24 impaired p53 activity by suppression of acetylation and increased interaction with MDM2 protooncogene. This impairment of p53 suppressed the hydrogen peroxide-induced apoptotic response in hepatocytes and resulted in upregulation of tumorigenicity in hepatocytes [46, 56].

It has been demonstrated that DHCR24 expression was upregulated in HCC cell lines and tissues from IFN nonresponders LC and HCC patients. In a recent work of Ezzikouri et al., a set of patients with HBV/HCV ongoing liver disease has been analyzed for serum DHCR24 antibodies using enzyme-linked immunosorbent assay. The serum anti-DHCR24 antibodies levels were significantly higher in patients with chronic hepatitis C (CHC) than in healthy controls and, interestingly, in early HCV-induced HCC than CHC or liver cirrhosis (LC) patients and in late HCV-induced HCC compared to early HCC-C patients, which demonstrates a stage-related overexpression of DHCR24. The merits gained by DHCR24 as a novel biomarker of HCV-induced HCC rely on the fact that the sensitivity of anti-DHCR24 antibodies detection was shown to be higher than that of other biomarkers (alpha-fetoprotein and protein induced by vitamin K absence or antagonist-II) and that DHCR24 was upregulated in HCV-positive, but not HBV-positive, tissues or HBV/HCV negative HCC specimens [57]. Other data show that DHCR24 enzymatic activity in the cholesterol transport process triggers the DHCR24 surface expression and that DHCR24 possess a binding site of 2-152a MAb that exerts anti-HCV activity and induce the downregulation of DHCR24 surface expression, shedding the light on the potential function of HCC-surface expressed DHCR24 as carrier of target antitumor agents [46, 58].

The importance of DHCR24 as a novel biomarker of interest rises from its usefulness for early detection of disease progression and specifically HCC. On the other hand, it might represent a new target for HCC therapy leaning on its property of binding to 2-152a MAb, which may be promising tool in the future for the HCC targeting approaches [46]. Nonetheless, there is still too much to learn about DHCR24 biology in the context of HCC, especially posttranslational modifications, how it could affect the HCV life cycle and the disease progression and the human population context, and how it determines DHCR24 expression and functional profile.

## 5. Conclusion

It is well known that HCV exploit the host lipid machinery to replicate and spread. The available data suggest that HCV-infected cells may become antiapoptotic and replicate efficiently to establish chronic infection through overexpression of DHCR24. Thus, the HCV-induced oxidative stress responsive protein DHCR24 may play a critical role in the pathogenesis of not only HCV persistent infection, but also associated liver diseases as steatosis, steatohepatitis, and cirrhosis. The role of DHCR24 in HCV-associated liver diseases lies in its aberrant expression altering programmed cell death pathways. The sustained expression of DHCR24 in response to HCV-induced oxidative stress results in suppression of nuclear p53 activity by blocking its acetylation and increasing its interaction with MDM2 in the cytoplasm leading to its degradation, which may induce hepatocarcinogenesis.

In conclusion, DHCR24 represents a potential marker of HCV-induced HCC development and might be a prospective target to HCC treatment in regions with high prevalence of HCV- induced HCC.

## Abbreviations

ALT: Alanine aminotransferase
AST: Aspartate aminotransferase
CAT: Catalase
CD81: Cluster of differentiation 81
CLDN-1: Claudin-1
Cyt c: Cytochrome c
DC: Dendritic cell
DC-SIGN: Dendritic cell-specific intercellular adhesion molecule-3-grabbing nonintegrin
DHCR24: 3*β*-Hydroxysterol Δ24-reductase
ER: Endoplasmic reticulum
GPX1: Glutathione peroxidase
GSR: Glutathione reductase
GST: Glutathione transferase
H_2_O_2_: Hydrogen peroxide
HBV: Hepatitis B virus
HCADS: HCV-associated dysmetabolic syndrome
HCC: Hepatocellular carcinoma
HCV: Hepatitis C virus
HMOX: Heme oxygenase
HO^•^: Hydroxyl radicals
HSCs: Hepatic stellate cells
IRES: Internal ribosomal entry site
MAMs: Mitochondria-associated ER membranes
MAPK: Mitogen-activated protein kinases
MDM2: MDM2 protooncogene, E3 ubiquitin protein ligase
NADPH: Nicotinamide adenine dinucleotide phosphate
NF-*κ*B: Nuclear factor kappa B
NK: Natural killer
NO: Nitric oxide
NO_2_: Nitrogen dioxide
NO_3_: Nitrate
NS: Nonstructural
O_2_^•−^: Superoxide anions
OCLN: Occludin
PON1: Paraoxonase 1
PP2A: Protein phosphatase 2A
PRDX: Peroxiredoxin
RNS: Reactive nitrogen species
ROS: Reactive oxygen species
S1R: Sigma-1 receptor
SOD: Superoxide dismutase
SR-BI: Scavenger member 1 receptor-class B
SREBF1: Sterol regulatory element binding transcription factor 1
TNF-*α*: Tumor necrosis factor-*α*
TXNR: Thioredoxin reductase
UTR: Untranslated region
VLDLR: Very-low-density lipoprotein receptor.
